# Testing standards for AI-based scores in automated essay scoring

**DOI:** 10.1371/journal.pone.0354680

**Published:** 2026-07-31

**Authors:** Rudolf Debelak, Matthias Ziegler

**Affiliations:** 1 Institute of Education, University of Zurich, Zurich, Switzerland; 2 MLO Lab, EPFL, Lausanne, Vaud, Switzerland; 3 Department of Psychology, Humboldt Universität zu Berlin, Berlin, Germany; University of Lahore - Raiwind Road Campus: The University of Lahore, PAKISTAN

## Abstract

Recent developments in the field of artificial intelligence and machine learning allow the wide application of large language models for the evaluation of written text and other non-numerical data. When applied in the context of psychological and educational assessments, such models can be used for assigning scores to essays and other types of responses. In contrast to classical tests, essays do not consist of test items, which leads to specific challenges in the evaluation of testing standards for scores obtained from AI models that differ from those observed for classical ability tests and personality questionnaires. To address these challenges, we discuss the evaluation of validity, fairness, and reliability for scores obtained from models of artificial intelligence in the context of automated essay scoring. We review existing methods, propose new methods, and further illustrate the reviewed methods with an empirical example based on the Hewlett Foundation data set on automated essay scoring. By applying the proposed framework to an evaluation based on a DistilBERT model, we find the model to be robust with sufficiently high internal consistency (Spearman-Brown coefficients in the range from .77 to .92). We further found empirical evidence for the validity of the evaluation model, but also indications for violations of fairness when comparing the human and AI scores across different topics. This study provides a standardized, replicable toolkit for researchers and practitioners to evaluate the psychometric quality of AI-based assessments.

## Introduction

Recent developments in the field of artificial intelligence (AI), such as the wide availability of large language models (LLMs) via models such as OpenAI’s GPT-4 [1], GPT-4o [2] and Google’s Gemini [3] have led to numerous applications in the field of psychological and educational testing. Prominent examples include the automatic scoring of essays or the use of LLMs to generate and evaluate items for educational assessments [4–11]. This line of work is sometimes summarized as Computational Psychometrics or Educational Data Mining [12,13]. The integration of these tools in psychological and educational assessments is the topic of an ongoing debate [14–20] and also includes applications such as automated item generation or the use of AI models to provide feedback. In this context, it is still a point of discussion whether AI models understand the text they process [21], which leads to the necessity of a rigorous evaluation of their output. Despite the numerous methodological innovations of this field, educational assessments based on AI still have to meet professional psychometric standards as they are outlined, for instance, in the Standards for Educational and Psychological Testing [22] or the Guidelines for Technology-Based Assessment [23]. Rich literature discusses the psychometric evaluation of multi-item measurement (for example: [8,24]). This concerns in particular an evaluation of AI-based scores with regard to the reliability, validity and fairness of their interpretation. Scores obtained from AI models use a different methodological framework than traditional testing scores, e.g., natural language processing methods, which do not use trivial scoring functions, such as the sum of correct responses, or a statistical function such as a maximum likelihood estimation to obtain a score. In this aspect, their evaluation differs from frameworks such as classical test theory (CTT) [25], factor analysis (FA) or item response theory (IRT) [26]. Instead, AI models are usually compared based on quantitative metrics in various benchmarks and based on their underlying training data.

This paper wants to review and discuss methods for checking the reliability, validity and fairness of the interpretation of AI-based test scores. By “AI-based scores,” we refer to any numerical evaluation that is given by a machine learning model, such as an LLM, to a human response to a task. Examples for such AI-based scores include, for instance, numerical scores provided by models such as BERT [27] or GPT-4o [2], or scoring systems based on numerical linguistic features such as e-rater [28]. However, we do not consider, for instance, verbal feedback, as it can be provided by generative AI models such as GPT-4o, but briefly discuss possible approaches for evaluating verbal feedback near the end of this work. In the empirical sections below, we use the term “predictions” when referring specifically to the output of a trained model, which we treat as synonymous with AI-based scores in this context.

Besides reliability, validity, and fairness, many additional testing standards are proposed in the literature, such as the standardization and usefulness of a psychological or educational test. However, we focus here on these three standards for two reasons: a) Reliability, validity, and fairness are usually considered as central for the evaluation of tests, and test scores that fail to be reliable, valid, or fair are usually considered problematic. b) The evaluation of these three testing standards usually relies on statistical frameworks, such as CTT, FA, or IRT, which are usually not directly applicable to all aspects of the evaluation of AI-based scores. Although one can still apply simple statistical methods for checking these standards, e.g., the calculation of correlations between AI-based scores and human evaluations as a simple measure of evidence for convergent validity, we will propose additional methods to complement these classical methods.

### Classical and AI-based scores

In the following, we distinguish between *reliability* as the consistency of scores across equivalent inputs and *robustness* as the stability of scores under minor, construct-irrelevant perturbations.

AI-based scores, in the sense we have outlined above, differ in several important ways from the scoring of CTT or most latent variable models. First, the input of these models is usually not considered as a set of relatively independent units (i.e., items), but more sequential in character. Second, systems for obtaining AI-based scores, such as essay scoring systems based on transformer models and other types of neural networks, are establishing a relationship between the input text and their scoring, which is highly non-linear, typically includes complex interaction effects and is generally significantly more complex than the relationship established in models of IRT or FA.

A practical limitation of the discussed and proposed methods is that they assume that the predicted score is numerical or categorical in nature. In principle, LLMs can provide verbal feedback to texts and documents, including essays; as a simple illustration of this point, readers might use online interfaces to LLMs to ask these models for feedback on the spelling or the style of an arbitrary text. For such evaluations, the proposed methods cannot be applied, and future research might discuss the evaluation of assessment standards for validity, reliability, and fairness for such evaluations.

In summary, we aim to make the following contributions to the literature: a) We provide a systematic discussion of methods for evaluating reliability, validity and fairness for numerical test scores that were obtained from AI models. b) While previous work [29] discussed general frameworks, this paper contributes specific, testable methodologies. c) We illustrate the application of the proposed methods in an empirical example, which is based on automated essay scoring with modern Transformer models [30].

In the following Table 1, we provide a summary of the proposed evaluation steps, and how they relate to traditional psychometric standards. This operational testing protocol translates abstract psychometric concepts into concrete, testable procedures for AI models.

**Table 1:** Proposed Operational Framework for Evaluating Psychometric Standards in AI Models. This framework translates traditional psychometric standards into actionable evaluation methods and quantitative metrics for AI-based scoring systems.

Psychometric Standard	Operational Definition	Proposed Evaluation Method	Quantitative Metrics	Expected Outcome
**Reliability (I):** Internal Consistency	Consistency of the model in assessing the construct across text partitions.	**Split-Half Analysis:** Divide input text into comparable halves (e.g., odd vs. even sentences) and correlate scores.	Pearson *r*, Spearman-Brown coefficient, Yule’s Q.	High correlation between halves (adjusted for length), indicating stability of the scoring function.
**Reliability (II):** Robustness	Resistance of the score to construct-irrelevant variance (noise).	**Input Perturbation:** Introduce minor, irrelevant noise (e.g., 10 random typos) and re-score.	Correlation between original and perturbed scores; Mean Score Difference.	High correlation (*r* > .90) and negligible mean score degradation.
**Validity (I):** Convergent Validity	Alignment of AI scores with the “Gold Standard” (human expert ratings).	**Human-AI Agreement:** Compare model predictions against human ratings in a held-out test set.	*Regression:* RMSE, Pearson *r*. *Classification:* Accuracy, F1-Score.	Agreement metrics comparable to or exceeding human-human inter-rater reliability benchmarks.
**Validity (II):** Construct Validity	Sensitivity to intended construct quality rather than surface features.	**Degradation Analysis:** Introduce meaningful errors (e.g., 50 typos /structural flaws) expected to lower quality.	Correlation drop; Mean score reduction.	Significant, systematic decrease in scores, confirming the model detects quality loss.
**Fairness:** Prediction Invariance	Equal model performance across relevant subgroups (demographic or topic-based).	**Subgroup Analysis:** Stratify test set by groups (e.g., Topic, L1/L2) and compare performance.	Δ Accuracy/RMSE; Mean Signed Error.	Comparable accuracy across subgroups; Zero or near-zero mean signed error (no systematic bias).

The rest of this paper is set up as follows: First, we provide an overview based on the standards of reliability, validity, and fairness, which is based on their definition in the AERA, APA, and NCME testing standards [22]. Each overview is followed by a review of existing and new methods on how these standards can be checked for AI-based scores. We then illustrate some of these methods using an empirical example and conclude with a discussion.

## Reliability

The testing standard of reliability pertains to the precision of measurement, ensuring that scores do not contain large measurement errors [22]. See, for instance, Martinková and Hladká [8], for a general introduction to reliability.

### Reliability of AI-based scores in traditional frameworks

Traditionally, reliability is assessed using coefficients like Cronbach’s α or split-half correlations in CTT, or via the precision of parameter estimates (e.g., standard errors) in structural equation modeling and IRT [8]. All these concepts share the assumption that the chosen parametric modeling framework provides an accurate description of the data.

In the evaluation of automated essay scoring systems, classical frameworks can sometimes be applied by treating different facets of text quality as “items” to estimate an average rating’s reliability. A similar approach was chosen by Fan et al. [31] to determine the reliability of personality scores obtained by automated systems.

A related challenge stems from issues such as rater drift and halo effects that may affect individual raters. Those challenges are traditionally also faced by human raters. To quantify these effects, one can assess the agreement of various raters – which could include both AI models and human raters – by calculating the interrater reliability of various raters, which can be achieved, for instance, by calculating the intraclass coefficient [32]. A review and discussion of intraclass coefficients was provided by Liljequist et al. [33], Koo and Li [34] and others. Measurement frameworks such as IRT [26] can be used to correct for these effects. Also see Martinková et al. [35] for an overview on flexible modeling of heterogeneity in reliability estimates across groups.

As Attali [36] notes, such measures of interrater reliability are not suitable for assessing the reliability of AI-based systems themselves, since they do not compare the outcome of such systems under different conditions, such as different prompts. Instead, they aim at assessing to which extent AI-based scores can replace human ratings. As the same author writes, many early evaluations (e.g., [37]) of automated essay scoring systems aimed at comparing the agreement between those systems and human raters with the average agreement of human raters.

In summary, we can observe the following methods for assessing the reliability of automated essay systems using traditional methods:

- Treat different outputs based on text partitioning that are provided by automated essay scoring systems as “items,” and apply classical measures from CTT or IRT.
- Treat AI-based scores as additional ratings and compare them with those of human raters.
- If scores from multiple essays written by the same authors are available, one can consider test-retest reliability. However, this approach needs to consider that the writing quality of texts written by the same author can depend on the writing task, including the genre of the text [38].

### Additional methods for assessing reliability

When evaluating the reliability of AI-generated scores, it is necessary to consider that such scores depend on characteristics of the input (i.e., the essay) as well as of the model (i.e., the rater). To evaluate this interaction, it is thus necessary to evaluate different parts of it. To this aim, we suggest several methods that aim at manipulating the input to learn about characteristics of the scores, and indirectly about the model as a rater.

As was already outlined, a simple concept for reliability is based on the thought experiment that test takers can take the same test twice, and that the resulting test score should be stable in such a thought experiment. In the context of LLMs and similar AI models, such a thought experiment can indeed be carried out, for instance, by giving the same prompt twice to an AI model and comparing the resulting output. However, it is usually possible to determine the fluctuation of the resulting output to the same input by setting suitable hyperparameters, such as the temperature or the seed parameter, and using suitable hardware. Using these technical settings, it is, therefore, usually possible to optimize this aspect of reliability under specific settings. In other settings, such as multi-cloud settings with variable hardware, AI models may produce non-deterministic outputs due to the inherent variability of the underlying infrastructure. In such cases, it is recommended to quantify the variability of the output by repeatedly providing the same prompt to the model and analyzing the distribution of its responses. This can be done by calculating the standard deviation or variance of the generated scores, or by visualizing the distribution of outputs. For the remainder of the discussion, we assume that our AI model is reliable in the sense that it will always provide the same output to the same input.

Another important aspect of reliability concerns the idea that reliability is measured by the precision of ability estimates. This idea is not directly applicable to AI-based scores such as numerical evaluations obtained in an automated essay scoring system. This has several reasons: First, AI-based scores are usually not based on parametric statistical theory, which prohibits a statistical estimation of measurement errors. Second, the input of such AI systems, such as written text, is of a different nature than the data used in CTT, structural equation modeling or IRT, which are usually responses coded as integer values. While we focus on automated essay scoring here, similar problems arise in other applications of AI in educational assessments, such as simulations or the evaluation of constructed response tasks. As alternatives, we suggest two methods for assessing the precision of an AI-based score that are based on similar ideas such as those outlined for the traditional frameworks.

Both proposed methods share the assumption that the AI model used for obtaining the score already provides predictions of satisfactory accuracy. This assumption can usually be tested using well-known approaches in AI, such as assessing the prediction accuracy in a test set. However, it is important to note in this context that accuracy should not be confused with reliability. Accuracy more closely resembles the notion of model fit in FA or IRT. We will demonstrate the calculation of prediction accuracy briefly in our example below. To evaluate the reliability, that is, the precision of an AI-based score, we check how much this score changes depending on (usually small) changes of the provided input which stems from the same respondent. This pertains to the following simple idea, which also underlies the reliability estimators in traditional frameworks: The observed input can be considered as a sample of a respondent’s behavior, and leads to an observed AI-based score that aims to assess the proficiency of a respondent to show a specific behavior, e.g., writing texts of high quality. If we assume that this proficiency is sufficiently stable – and this assumption is commonly made in psychological and educational assessments -, the AI-based score should remain relatively stable regardless of the provided input. This leads to the following two methods for assessing reliability for AI-based test scores: First, we can divide the provided input in several parts, and obtain scores for each part using our AI model. A measure that summarizes the stability of these scores over the different parts can thus be considered as a measure of the internal consistency of the scores provided by the AI model – it evaluates whether the model provides consistent scores across different sections of the same text. Variations of this method have already been proposed in the context of automated essay scoring systems. Attali et al. [36] discussed an analogous method in the context of automated scoring systems that are based on features (e.g., word length). Here, the reliability of these features was estimated by calculating them for the odd and even sentences of essays and calculating the stepped-up correlation between both features. A similar method was proposed by Fan et al. [31] in the context of personality assessment. Here, the reliability of the prediction was estimated by the correlation between the predictions of the first and second half of the text.

It is well-established that essay length can correlate with both human scores and scores generated by automated essay scoring systems (e.g., [39]), as will also be shown in the empirical illustration below. It needs to be expected that applying a split-half method leads to a drop in the scores for the shorter halves. The purpose of the proposed method is thus not to test for a correlation between essay length and overall score, but to assess the consistency of the scoring function across different parts of the text. It accounts for a possible effect of the essay length on the model score by comparing parts of comparable length.

Another possible disadvantage of the presented method for quantifying the internal consistency of a scoring method is that it might result in unrealistic texts. For instance, extracting every other sentence from a text can create overly artificial texts. As an illustration, the reader can imagine two texts that only consist of the odd and even sentences of this manuscript. Predictions based on such unrealistic texts may not be representative for the scoring task for which reliability should be estimated. In other contexts, such as the analysis of unordered word lists, this approach can be less problematic. As an anonymous reviewer noted, this approach could even account for changes in the text structure that might affect the text ratings. A closer investigation of this method is left as a topic for future research.

A second method is related to approaches that are used for testing the robustness of an AI model. In this method, the input of the AI model is slightly changed, for instance by inserting a few characters or spaces. While the first method aims at evaluating the internal consistency, this method aims at evaluating the robustness of the scores – it assesses whether the model’s score is robust against minor changes to the essay that should be insignificant. Conceptually, adding such additional information can be compared to adding unsystematic variance to items in a psychological or educational test. The stability against such changes can be evaluated by applying such changes multiple times. This second method is related to the idea of adversarial examples in deep learning [40], which aims at checking the robustness of model predictions against minuscule changes in the input, which are assumed to be unrelated to what the AI model is aimed to assess.

For instance, if we consider an essay scoring system that is aimed to assess the overall style or consistency of an essay, it makes sense to include small typos and evaluate their effect on the assessment. If we consider an essay scoring system that assesses the spelling of an essay, it might make sense to replace individual words by synonyms and evaluate the effect of this change on the rating. The empirical example below aims to assess the overall writing quality, as defined by the pooled judgment of human raters.

In general, the definition of which changes are minuscule or irrelevant must be informed by a theory of writing. In a model assessing overall writing quality, extra spaces or punctuation problems can be theorized to have a more minor impact compared to errors in structure or argumentation. This method aims to test whether the model’s scores and sensitivity align with such hypotheses and is related to broader efforts in stress-testing AES and assessing their vulnerability to ‘gaming’ behaviors, where more targeted and substantial manipulations are employed to test system integrity [41,42]. If, for instance, a few typos cause a drastic score change, the model may focus too strongly on such features, which could be considered as a threat to the validity of its scores. The sensitivity of a given model to such changes is, of course, dependent on the specific model architecture and its training data. The outcome of this analysis is thus not a universal estimate for reliability but a statement on the behavior of the scores of a specific model.

A variation of this method, which is available with generative AI models such as OpenAI’s GPT-4o or Google’s Gemini, is the inclusion of small changes in the prompt, that is, the instruction to the generative AI model, and the evaluation of their effect on the score. A similar method was proposed by Attali [36], who compared automated essay scoring models based on different prompts (here: writing instructions to students) to parallel test forms in CTT. Following this author’s reasoning, one might estimate the reliability of these parallel test forms via the Spearman-Brown formula, a classical approach of CTT. In summary, the first proposed method, aiming at evaluating internal consistency, consists of the following steps:

- Divide your input, e.g., text, in two or more parts of comparable length. Calculate the AI-based score for each part of the input.
- As an estimate of internal consistency, calculate a measure of stability between the scores for all parts. For instance, if we divide the input into two parts, we could calculate the correlation between the numerical scores over the complete sample as such a measure. For more than two parts, adaptations of reliability measures from CTT, such as the Spearman-Brown formula, could be an alternative approach. Speer et al. [43] note that the interpretation of this correlation as a measure of reliability requires the assumption that both test halves are parallel.

The second proposed method, aiming at evaluating the robustness of an AI model, can be summarized as follows:

- Make small changes to the input, for instance, by inserting typos. The number of changes might be fixed or varied based on the text length. As outlined above, it is crucial that these changes are unrelated to what the model aims to assess. In models of generative AI, such as GPT-4o or Gemini, one could alternatively vary the prompt that leads to a numerical evaluation of the essay.
- The outcomes of the different changes or different prompts are considered as parallel forms of a test. One can now estimate the reliability of the test by applying traditional measures from CTT, such as the Spearman-Brown prophecy formula. For instance, in the case of two forms, one can calculate the correlation between the two evaluation scores before and after the changes and apply the prophecy formula.
- As a further enhancement, repeat this procedure multiple times with different types of changes (e.g., inserting typos, replacing names with synonyms) and obtain an estimation of the typical change of the ratings (i.e., the average change and its variance) for each type of change.

Since the second method also requires a theory on which changes to the inputs or prompts are inconsequential, it also evaluates an aspect of construct validity. We will discuss this aspect later.

The second method can be further adapted by gradually inducing more changes, e.g., more typos, and evaluating their effect on the overall score. This enhanced procedure would lead to a range of values for measuring the robustness as a facet of reliability instead of a single number for each type of change. If the changes to the text are too extensive to expect no effect of the score, a similar method can be used for assessing the validity of the score. We discuss this topic next.

**Summary of Reliability Evaluation:** In summary, assessing the reliability of AI-based scores requires moving beyond traditional parametric error estimation. We operationalize AI reliability through two complementary approaches: evaluating internal consistency via split-half text partitioning, and assessing robustness by introducing minor, construct-irrelevant perturbations (e.g., minor typos) to ensure score stability.

## Validity

Validity refers to the degree to which empirical evidence and theory support the intended interpretation of test scores ([22], see [8] for a practical application). Here, we focus on *validity* as the sensitivity of scores to meaningful differences and their alignment with the intended construct.

### Validity of AI-based scores in traditional frameworks

Traditionally, validity evidence is gathered from multiple sources, including test content, response processes, internal test structure, relations to external variables, and the consequences of testing [22]. In traditional settings, this involves structural evaluations using latent variable models (e.g., FA or IRT) to confirm construct validity, or correlational analyses to establish convergent and discriminant validity [44,45].

When applied to AI-based scores, particularly automated essay scoring, some of these traditional forms of validity evidence require adaptation [31,36,46]. For example, evidence based on response processes translates to investigating whether an AI model’s ratings are driven by construct-relevant features (e.g., argumentative structure) rather than construct-irrelevant artifacts (e.g., superficial formatting). Similarly, evaluating the relation to external variables involves measuring the degree to which AI-based scores predict relevant outcomes or correlate with independent human expert ratings. Finally, while a full analysis of the intended and unintended consequences of testing [47] is outside the methodological focus of this paper, it remains an essential consideration for the responsible deployment of any AES system to ensure it does not systematically disadvantage specific test-takers [22]. Other forms of validity evidence, such as using correlations as part of the evidence for convergent and discriminant validity, can be applied as in a traditional test analysis.

### Additional validity evidence

Some sources of validity evidence, in particular the response processes, are independent from the applied measurement framework. In the context of applications of AI models, one validity aspect of test content can be evaluated by checking the representativeness of the used training, validation and test data sets. Although this is not evidence for content validity in the traditional sense, the representativeness of the training data ensures that the model learns a grading function that is applicable in the entire target population. In the rest of this section, we therefore focus on validity evidence based on the internal structure of the test and the relation to variables external to the test.

Williamson et al. [29] suggested several numerical criteria that could be evaluated and compared, for instance: a) The agreement of AI scores with human ratings, b) the degradation from human-human agreement (i.e., the difference between human-AI agreement and interrater agreement), where such information is available, c) the standardized mean score difference between AI and human ratings. These evaluations correspond to evaluating the convergent validity of AI-based systems. Furthermore, they suggested the group-wise evaluation of external and within-test relationships, which corresponds to the idea of evaluating prediction invariance of the group level.

Using a similar perspective, we can propose additional methods for evaluating validity based on the methods outlined above. If AI models are used to predict external criteria, such as the rating of human raters for texts or pictures, the accuracy of this prediction can serve as evidence for a relation between the test score, as provided by the AI model, and an external criterion, such as human ratings. This idea enhances the classical criteria for convergent, discriminant and criterion validity outlined in the previous section. In computer science, this type of investigation is usually summarized as an evaluation of the model generalizability to new data by evaluating its predictive accuracy in training, validation and test data. In this context, usually two types of prediction models are distinguished: While regression models aim at predicting continuous variables, classification models aim at the prediction of categorical variables. Both model types differ in the metrics that are usually used for assessing the prediction accuracy. Usual metrics to quantify such a relation typically include the accuracy, the F1 score, the sensitivity, and specificity in classification models, as well as the root mean square error (RMSE) in regression models. To understand the F1 score, one can imagine a diagnostic test that results in a positive or negative outcome. In this scenario, “precision” indicates how often a positive outcome is truly correct (avoiding false positives). “Recall” indicates how many true cases were caught (avoiding false negatives). The F1 score is the harmonic mean of precision and recall; it is essentially a single number that rises only when both are good.

This approach for evaluating this aspect of validity is based on the simple idea that the AI score is treated as an outcome of the assessment whose relation to external variables can be directly investigated. This evaluation can be carried out in the training set, that is, in the data that were used for training the model, but also in a test set that was not used for training. In this approach, we treat the AI-based scores as any other score.

A related, but distinct question pertains to explaining how an AI model, for instance for essay scoring, obtained its prediction. This question is related to the second mentioned type of validity evidence, namely, whether the score of the AI models corresponds to its intended interpretation. Such tools are provided by the methods of explainable AI, or interpretable machine learning [48]. While an overview of all available methods is outside the scope of this paper, available methods from the field of natural language processing entail LIME values [49] and SHAP values [50]. For individual data points, these methods help to explain how changing individual features, such as tokens in the context of NLP models (e.g., words or word parts), affect the prediction of the model. For instance, if an AI rating aims to evaluate the spelling of a text, SHAP and LIME values should indicate which words or word parts in a text affect the spelling assessment. Ideally, they would mark all spelling errors, which should affect this assessment negatively. We do not illustrate this method in the empirical example, which is based on essay scoring, later in this text, since it is not clear for this example how specific tokens would relate to the overall rating of the text. In this sense, content validity could be limited in some use cases of essay scoring models. An example application can still be found in the Python code that accompanies this text.

As a variation of this idea, we can induce changes that are related to the trait the assessment aims to measure. For instance, when evaluating an essay scoring system that focuses on spelling, inserting typos should typically reduce the overall rating of an essay. A second aspect concerns the size of the observed changes. Insignificant changes should have insignificant effects on the rating, while significant changes should have significant effects in the desired direction. Therefore, the extent of the changes to the input texts and its expected effect on the ratings determines whether this method is used to assess the robustness, as a facet of reliability, or the validity of the AI-generated scores. A similar point related to construct overlap and the interpretation of correlations as reliability and validity is made by Speer et al. [43].

Optionally, one could plot the range of the observed score in a visualization that is reminiscent of confidence intervals. This approach of inducing changes is conceptually related to the ideas underlying SHAP values (cf. [48]).

Some methods of explainable AI can also be used to investigate the internal structure of AI models. Under the perspective of getting sources of validity evidence, these methods can be seen as belonging to the third type of validity evidence. For instance, software such as TensorBoard, which is part of TensorFlow [51], can help to visualize how information is processed in neural networks. These methods for visualizing the internal structure of neural networks can be considered as a potential future direction for validity research. They could be used to investigate whether individual neurons or neural layers specialize in detecting features that align with specific aspects of the scoring, such as scoring. This type of methodology is currently largely unexplored, therefore we focus on more readily available methods in this paper.

Another method that may help to explain the prediction of an NLP model is to check the scoring of (short) texts for which the true grading is known, but which are not part of a model’s training data. These might also include extreme cases; in the context of essay scoring, such a case might consist, for instance, of an essay with no language errors that is written on the wrong topic. A similar approach was evaluated by Ding et al. ([52]). This method aims at checking whether a given AI model shows undesired or unexpected predictions in specific cases. This method may also help to confirm whether the model applies heuristics that may lead to incorrect predictions in specific cases. Again, this evidence helps to understand the scoring of an AI model, and whether it agrees with the intended interpretation. In summary, the presented methods for checking the validity of AI-based scores can be summarized as follows:

- To assess the validity of an AI model, its predictions can be compared against external criteria. When the criterion is a continuous variable, such as human ratings, the correlation coefficient or the root mean squared error can be used to quantify the accuracy of the AI’s predictions. For categorical criteria, metrics like accuracy, sensitivity, specificity, and the F1 score are appropriate, especially when dealing with binary classifications.
- Use methods of interpretable machine learning and explainable AI, such as LIME and SHAP values, to investigate the relation between individual features and the model prediction. For instance, do such methods indicate correctly that words containing spelling errors decrease the overall quality of a text? Alternatively, investigate the effects of inducing small changes for which the expected effect on the evaluation is known. Using the same example, does inserting more spelling errors reduce the rating of the text?
- Investigate the behavior of specific neurons or specific layers as part of the investigation of the internal structure of an AI model. This method could, in theory, help detect whether individual neurons or layers specialize on aspects of essay scoring, but it is relatively unexplored.
- Evaluate predictions for artificial cases where the true evaluation is known. For instance, does the model detect correctly if a text contains flaws in its argumentation and structure, or does it rather focus on obvious errors in spelling, grammar and punctuation?

A further possible extension arises if ratings from multiple AI models and multiple raters are available. Here, one could follow a traditional approach by applying a multitrait-multimethod analysis (e.g., [53]) that investigates the pairwise correlations for raters and AI models, or a more advanced CTC(M-1) model [54].

**Summary of Validity Evaluation:** In summary, validating AI-based scores requires a multi-faceted approach. This includes comparing AI predictions against external criteria (e.g., human ratings) to establish convergent validity, using explainable AI (e.g., SHAP/LIME) to verify the relevance of modeled features, and stress-testing the model with targeted, construct-relevant text manipulations (e.g., degradation analysis) to ensure the model reacts appropriately to true quality loss.

## Fairness

Fairness is not uniquely defined. Following the Standards for Educational and Psychological Testing ([22], p. 50), “a test that is fair … reflects the same construct(s) for all test takers, and scores from it have the same meaning for all individuals in the intended population; a fair test does not advantage or disadvantage some individuals because of characteristics irrelevant to the intended construct.” Such characteristics typically include gender, ethnicity, socioeconomic status, or cultural background [22]. Important aspects include the fairness with regard to the treatment during the testing process, the absence of measurement bias, the access to the measured constructs, and the validity of the intended test interpretations. Although not all of these can be evaluated using statistical measures, an important group of statistical evaluations is summarized under the term of machine-learning measurement bias [55] or the assessment of prediction invariance [56,57].

In practical evaluations of fairness, it is crucial to move beyond single-axis fairness analyses, since it is often not sufficient to investigate fairness for specific groups defined by only gender or only language. Instead, it is crucial to consider violations of fairness that affect individuals at the intersection of these groups, e.g., female Spanish-language learners.

Another important aspect of the evaluation of fairness is the discussion of individual differences. AI models must not only be fair at the level of groups and their intersections, but they must also account for individual differences that are not relevant to the constructs that are being assessed. For instance, an automated essay scoring system must account for differences in writing styles and cultural backgrounds.

It seems important to note that AI research is usually based on a related concept of fairness, which aims at comparing the accuracy of prediction of an AI model in various groups of interest. The basic idea is to define groups of interests and to compare the predictive accuracy between these groups. For a more detailed discussion of fairness issues in the context of machine learning, see, for instance, Barocas et al. [58] or, in the context of psychological assessments, Goretzko and Israel [59].

There are important aspects of fairness that need to be evaluated before the application of LLMs and other models of machine learning. The first aspect concerns the presence of biases within a pre-trained LLM [60], which was recently discussed in the context of essay scoring by Johnson and Zhang [61], and in the context of NLP models, by Hovy and Prabhumoye [62]. The second aspect concerns the question of whether the data used for training or fine-tuning a machine learning model can be considered as representative for the underlying population. As Schaller et al. [63] argue, training data that are not representative with regard to demographic background variables or cognitive ability can lead to biases or low accuracy in populations that are not represented in the training data.

### Evaluating fairness

Statistical methods to detect violations of fairness typically entail methods for the detection of violations of measurement invariance and for the detection of predictive bias. Methods for detecting violations of measurement invariance could be useful in modeling approaches that use latent variable models for developing a joint model for the numerical ratings of human and AI-based raters. Statistically, this can be achieved by a model of IRT, e.g., a multi-facet Rasch model, where the various raters correspond to items, and the essays to persons [64–67]. Conceptually, the basic idea is that all raters aim to assess the same latent trait, but might differ in their strictness (corresponding to the item difficulty) and, depending on the used model, their discrimination and other psychometric characteristics.

While methods of measurement invariance aim at detecting differences in psychometric characteristics of the test items, methods for detecting predictive bias aim at differences in the regression model used for the prediction of external criteria [56,57]. In contrast to methods of measurement invariance, these methods can be used to compare the accuracy of an essay scoring model between various relevant groups of essays. Similar to the concept of group fairness ([68]; see [69] for a critique), they aim at checking whether there are groups of respondents that are systematically disadvantaged by a model. This approach was also advocated by Tay et al. [55], who compared it to measurement invariance. They suggested the following four approaches for detecting differential model accuracy, which they also call machine-learning measurement bias:

- **Evaluation based on the score level**: Sample individuals from different relevant groups with the same observed rating score, which serves as a proxy for the true score. In the context of essay scoring, these could be individuals from different groups of gender, different language groups, etc., whose essays received the same score by human raters. Let the score of their essays be predicted by a machine learning model, and check for systematic differences in the predicted scores.
- **Evaluation based on the distribution level**: Sample individuals from different relevant groups with a similar distribution of the rating scores, e.g., with the same mean and variance. As with the first approach, obtain a prediction of their score by the machine learning model, and check whether the distribution of the predictions is comparable over different groups.
- **Evaluation based on the predictive accuracy**: Evaluate the predictive accuracy of the machine learning model for subgroups of interest. In contrast to the first point, “Evaluation based on the score level,” this point does not concern the investigation of systematic differences between the predictions in various subgroups. The focus of this evaluation is whether the predictions are equally accurate in all relevant subgroups.
- **Evaluation based on models for the score level and the score predictions**: In a first step, use a simple model, e.g., a linear regression model, to predict the score predictions obtained from the machine learning from the observed human ratings. In a second step, evaluate whether this model is stable for different relevant subgroups. A similar, well-known approach based on moderated regression models was used by Cleary [70].

Expanding on the argumentation of Tay et al. [55], the concept of prediction invariance can be generalized in a straightforward way to scores from non-linear regression models, such as AI models. A similar perspective is taken by Johnson et al. [20] and Johnson and McCaffrey [71] and Williamson et al. [29], who discussed several methods for evaluating fairness in the context of essay scoring systems. Williamson et al. [29] focused on the question of whether it is fair on the level of relevant subgroups to replace a human rater by an AI score. Under this perspective, violations of fairness might occur if the AI model disagrees with human raters for specific subgroups (e.g., female students), or gives systematically higher or lower ratings than human raters. Johnson et al. [20] discuss different perspectives on fairness in AI. For applications in psychology and education, they discuss *fairness based on separation* and *fairness based on sufficiency*. To illustrate these terms, we assume that we want to check an essay scoring system, which aims to assess verbal intelligence, for fairness with regard to gender. In a non-technical language, fairness based on separation means in the context of this example that the essay score obtained by AI is independent from, and thus uncorrelated to, gender for persons with the same level of verbal intelligence. Fairness based on sufficiency, on the other hand, means that the true verbal intelligence score is independent from, and thus uncorrelated to, gender for persons with the same predicted essay score level. Johnson et al. [20] also suggest statistical tests to detect violation of these types of fairness which are however based on additional modeling assumptions.

In summary, a fundamental check of the fairness of AI models can be summarized by the following steps:

- In a first step, divide the sample into groups of interest, for instance based on groups of categorical covariates. In metric covariates, create discrete groups based on the observed values, for instance age groups.
- In a second step, train or fine-tune the AI model. Evaluate and compare its accuracy for each group, that is, for each relevant subpopulation obtained in the first step. This assessment includes a) the evaluation of the predictive accuracy, b) the evaluation of whether the model provides systematically lower or higher scores than an AI-based model, or whether there are any systematic deviations of the AI-based scores from human ratings.

This evaluation can be further enhanced with specific metrics and visualizations that compare the model performance between different groups. These methods can be complemented by other, more traditional methods for checking the fairness of AI-based scores, which are based on a) modeling the rating behavior of human and AI-based raters via models of IRT in a first step, and b) checking its invariance across relevant sub-groups. This evaluation aims at checking the invariance of the overall rating behavior of all raters. Further, specific statistical tests for separation and sufficiency fairness can be conducted to evaluate independence assumptions between the predicted score, the true construct or ability, and person covariates. However, these tests might require additional modeling assumptions, therefore we do not focus on them here.

**Summary of Fairness Evaluation:** In summary, our framework for evaluating AI fairness centers on establishing prediction invariance. This involves systematically comparing model accuracy, error distributions, and score biases across relevant demographic or topic-based subgroups to ensure that the AI model does not systematically disadvantage any specific group of test-takers.

## An illustration with empirical data

As a next step, we illustrate several of the methods outlined above with an empirical data set. We showcase these steps using the training data from the Automated Essay Scoring Competition hosted by the Hewlett Foundation on Kaggle, which are available online (https://www.kaggle.com/competitions/asap-aes/data) and were also used for illustration in several other studies on automated essay scoring (e.g., [39]). The complete data set consists of eight essay sets with overall 12976 essays written in English. In each essay set, the essays were written by human students based on a specific prompt. A list of prompts is given in S1 Appendix.

All essays were rated by at least two human raters according to guidelines which differed slightly for each prompt, leading to at least an overall score, which serves as target value. Furthermore, all personally identifying information was removed from the published texts. A limitation of this public dataset is the lack of information on the human rater assignment process. It is unclear whether the same human raters scored all essays, which makes the interpretation of the observer inter-rater reliability problematic. Therefore, the findings of the reported analysis should be seen as an illustration of the methods rather than a definitive assessment of the scores provided by this AI model.

We split the 12976 essays into a training set of 7783 essays (60%), a validation set of 2596 essays (20%), and a test set of 2597 essays (20%), which are all stratified by the essay topics. An important practical restriction of this data set is that there are no known person covariates such as age or gender, so it is not possible to evaluate fairness with regard to such covariates. Further, it is unclear whether the two human raters per essay were the same raters for all essays or not, but it seems plausible that the raters differed for the individual essays.

After suitable preprocessing, the target value takes values between 0 and 1. In the resulting data set, at least two modeling approaches are conceivable: First, one could treat this problem as a regression problem, and aim to predict the specific target value. Second, one could frame this problem as a classification problem instead, by first dichotomizing the target value. In this problem, we would just be interested in whether an essay belongs to a specific group, e.g., the group of essays of high or low quality. Below, we demonstrate the evaluation of validity, reliability and fairness for both regression and classification problems. When the problem was framed as a classification problem, a threshold of .5 was used to distinguish between essays of high and low quality.

In the following, we assume that suitable preprocessing was carried out (e.g., the removal of any special characters) and that we have fitted a machine learning model to solve the classification or regression problem. In this demonstration, a DistilBERT model [30] that was pretrained on an English corpus was used. A Jupyter notebook that allows a replication of these analyses is available in the supplementary materials at https://osf.io/nr8he/ and as a Google Colab under the following link, although the exact results per run may differ slightly because of small differences in the fine-tuned models: https://colab.research.google.com/drive/1G-vBsGBxrZPV8PYxzHelnXaLw_fPNims?usp=sharing. This model is an encoder-based transformer model and leads to a numerical prediction of a target score. In principle, one could also use decoder-based transformer models such as GPT-4o [2] to get verbal or numerical evaluations of these essays. In the following sections, we only assume that a numerical score or label is predicted for each essay, but it is not important which type of model led to this prediction.

### Treatment as a classification problem

We start our discussion with the treatment as classification problem, that is, we simply try to predict whether an essay gets a “high” or “low” (or, alternatively, “pass”/“fail”) overall score. From a technical perspective, this is the simpler approach, although the dichotomization of continuous variables cannot be recommended in general. We used cross-entropy as a loss function, which is technically comparable to using maximum likelihood estimation of the model parameters.

We first check the overall agreement between the predicted and the true classes in the validation dataset as an indication of construct validity. In practice, the prediction accuracy can be assessed by multiple criteria, such as the sensitivity and specificity, the F1 score, the classification accuracy, and others. In the essay scoring problem, we obtained a loss of .27 in the training data and of .44 in the validation data after training for three epochs. In the validation data, we observed an accuracy of .85 and an F1 score of .84, which indicate a sufficiently high accuracy for the purposes of this illustration. In a later subsection, we will further investigate the accuracy for different topics of student essays in the training, validation and test data sets.

#### Assessing reliability.

A first approach for checking reliability is based on the basic idea that an AI model should give comparable scores for different parts of the text, if these scores are meant to represent the same underlying latent characteristics, such as the ability to write with correct spelling and grammar. In the context of essay scoring, there might be criteria where different parts of the texts might not represent the same latent characteristic. For instance, the style of an essay might change during the text. In such instances, the proposed split could be problematic as an estimate of the reliability of the AI model. We therefore determine the scores for different parts of the text. In our example data, we counted the number of sentences in each text, and then split each text into two parts that both contained a similar or equal number of sentences. Very short texts that consist of only one sentence are omitted in this analysis. For each half, we obtained a score prediction, and compared the predicted score for the first and second half of the text. The internal consistency of the predictions was evaluated using a 2x2 contingency table. For each text, the columns of the table represent the predicted outcome (positive or negative) based on the first half, while the rows represent the predicted outcome based on the second half. Each cell thus contains the frequency of texts for each combination of first-half and second-half predictions. The agreement between the predictions of each half can again be calculated by a suitable correlation coefficient, such as Yule’s Q or Cohen’s Kappa. In the following, we calculated Yule’s Q and report analytical confidence intervals (CIs).

In the example data set, we observe the results reported in Table 2 using the example code given in the online supplementary material.

**Table 2:** Confusion Matrices for the Predictions for both Text Halves.

		Prediction 1st Half					
		Training Data		Validation Data		Test Data	
		Neg. Pr.	Pos. Pr.	Neg. Pr.	Pos. Pr.	Neg. Pr.	Pos. Pr.
Pr. 2nd Half	Neg. Pr.	60.12%	14.32%	62.21%	13.72%	60.61%	15.05%
	Pos. Pr.	6.8%	18.76%	6.46%	17.61%	6.45%	17.89%

*Note.* Neg. Pr. = Negative Prediction; Pos. Pr. = Positive Prediction. Higher values on the diagonal (Neg./Neg. and Pos./Pos.) indicate greater consistency between the scoring of the first and second halves of the text.

This leads to a Yule’s Q coefficient of *Q* = .84 (95% CI: [.82; .86]) for the training data, *Q* = .85 (95% CI: [.82; .88]) for the validation data and *Q* = .84 (95% CI: [.80; .87]) for the test data. After applying the Spearman-Brown prophecy formula, we obtain values of *Q* = .91 (95% CI: [.90; .92]) for the training data, of *Q* = .91 (95% CI: [.90; .93]) for the test data, and of *Q* = .92 (95% CI: [.89; .93]) for the validation data. These values, especially those for the validation and test data, can be interpreted as measures for the model’s internal consistency. For an additional analysis, one could use alternative criteria to split the text in two halves. Overall, these coefficients can be interpreted as satisfactory when interpreted as split-half coefficients. An interesting finding is that, in the example at hand, most of the predictions are negative for the individual test halves, although they are generally positive for the complete essays. This might indicate a tendency of the model to give worse grades to shorter essays. As was already discussed, such an effect was already reported for human as well as automated scores. In addition, differences between the ratings of text halves could reflect genuine differences in text quality between the text halves. These explanations seem plausible in the context of this empirical example, and we therefore consider these coefficients as robust measures of the model’s reliability.

A second facet of reliability is the robustness of the evaluation. A group of possible evaluations concerns the question of whether insignificant changes of the input texts have no significant effect on the AI scores. In the context of this illustration, we induce small errors, i.e., typos, in the essays of the data set (i.e., training, validation and test data) and evaluate whether these changes lead to an insignificant reduction of the predicted scores. In the example presented here, ten random letters were inserted at random positions in each text, and the prediction of the model before and after this change was investigated. The changes in the probabilities for a positive outcome are presented in the following Table 3 for the training, validation and test data sets. For all three data sets, we observe Yule’s Q coefficients that are larger than .99, indicating a strong correlation of the predicted score with and without 10 typos. However, we also observe that the inclusion of typos overall reduces the predicted score of the essays. This observation helps to understand how the algorithm scores and thereby provides further insights into the score’s validity.

**Table 3:** Confusion Matrices for the Predictions with (Columns) and without (Rows) 10 Typos.

	Training Data		Validation Data		Test Data	
	Neg. Pr.	Pos. Pr.	Neg. Pr.	Pos. Pr.	Neg. Pr.	Pos. Pr.
Neg. Pred. (No Typos)	37.75%	.33%	36.48%	.19%	35.96%	.12%
Pos. Pred. (No Typos)	5.51%	56.40%	7.47%	55.86%	8.93%	54.99%
Agreement (%)	94.15		92.34		90.95	
Net Score Shift	+5.18		+7.28		+8.81	
Yule’s *Q*	> .99		> .99		> .99	

*Note.* Neg. Pr. = Negative Prediction; Pos. Pr. = Positive Prediction.

Diagonal cells represent agreement (robustness). Off-diagonal cells represent disagreement.

Agreement = sum of diagonal cells. Net Score Shift = % shifted from positive to negative

minus % shifted from negative to positive (in percentage points).

### Assessing validity

To further assess the validity of the rating, we investigated the effect of a more drastic change of the essay texts by inserting 50 typos. More advanced options, which were suggested by an anonymous reviewer, could include other types of small errors, such as using the wrong word, word repetitions, or word omissions. This change was expected to result in ratings that would have a smaller correlation with the original ratings, and would be overall more negative.

The changes in the probabilities for a positive outcome are presented in the following Table 4 for the training, validation and test data sets. For all three data sets, we observe Yule’s Q coefficients over .99, indicating a very strong correlation of the predicted score with and without 50 typos. As before, we also observe that the inclusion of typos overall reduces the predicted score of the essays.

**Table 4:** Confusion Matrices for the Predictions with (Columns) and without (Rows) 50 Typos.

	Training Data		Validation Data		Test Data	
	Neg. Pr.	Pos. Pr.	Neg. Pr.	Pos. Pr.	Neg. Pr.	Pos. Pr.
Neg. Pred. (No Typos)	37.83%	.15%	36.86%	.08%	36.62%	.15%
Pos. Pred. (No Typos)	20.4%	41.62%	22.38%	40.68%	22.6%	40.62%
Agreement (%)	79.45		77.54		77.24	
Net Score Shift	+20.25		+22.30		+22.45	
Yule’s *Q*	> .99		> .99		> .99	

*Note.* Neg. Pr. = Negative Prediction; Pos. Pr. = Positive Prediction.

Agreement = sum of diagonal cells. Net Score Shift = % shifted from positive to negative

minus % shifted from negative to positive (in percentage points).

For specific criteria, such as the detection of spelling and grammar errors, it might also make sense to use methods from interpretable machine learning, such as LIME and SHAP values. In general, these methods are designed to determine which features are relevant for obtaining a specific outcome. In the context of essay scoring, such values indicate which tokens, or word parts, affect the predicted score of an essay. A useful application of LIME and SHAP values requires that there is a theoretically meaningful relationship between the individual features and the dimension the essay scoring model is trying to assess. If the score aims, for instance, to evaluate the overall level of spelling and grammar, such values should detect and mark spelling errors in the essay as an evidence that the interpretation of the AI score is valid. Since the essay score aims at evaluating the overall quality of the essay, we do not apply LIME and SHAP values in this example.

#### Assessing fairness.

For assessing the fairness of the AI scoring, we focus on the evaluation of two related criteria that both depends on the availability of a categorical person covariate with regard to which we want to evaluate prediction invariance. The first criterion concerns the requirement that a) the prediction accuracy should be sufficiently high for all relevant groups of essays, and the second criterion concerns the requirement that b) the prediction accuracy should be at a comparable level for all relevant groups of essays. In a non-technical language, the first criterion demands that the AI score should be sufficiently predictive for all relevant groups of essays, and the second criterion demands that there should be no significant differences in the prediction accuracy. In practice, the prediction accuracy can be assessed by multiple criteria, such as the sensitivity and specificity, the F1 score, the classification accuracy, and others, which can be applied to evaluate the fairness of an AI score. As a third criterion for bias, we further demand that there should be no systematic bias for any relevant group of essays. That is, the numerical AI scores should be, on average, comparable to those given by human rater for all relevant groups of essays, and there should be no group where the AI model scores systematically higher or lower than the human raters.

Due to the absence of demographic data in the used dataset, a traditional fairness analysis across protected groups (defined by gender, ethnicity or similar characteristics) is not possible. To illustrate our methodology, we therefore focus in the following on differences of model performance and bias across subgroups defined by essay topics. This analysis should not be interpreted as a substantive evaluation of test fairness.

In the example data set, we define the relevant groups of essays by the topics on which the students should write. To check the first two criteria, we again obtain contingency matrices. In contrast to the evaluation of reliability, we are now interested in whether the agreement between human raters and the predictions of the AI model is comparable for all topics. The results for the training and validation data sets are presented in Tables 5 and 6.

**Table 5:** Confusion Matrices per Essay Topic which Compare the Observed Ratings with the Predictions from the AI Model. Predictive Performance was Strong across Semantic Domains, with Individual F1 Scores for the Test Set Ranging From .75 (Topic 2) to .95 (Topic 1), Resulting in a Macro-F1 Score of .87.

		Training Data		Validation Data		Test Data	
		Neg. Pr.	Pos. Pr.	Neg. Pr.	Pos. Pr.	Neg. Pr.	Pos. Pr.
Topic 1	Neg. Rating	13.66%	2.81%	9.8%	6.16%	8.68%	7.28%
	Pos. Rating	.84%	82.69%	3.64%	80.39%	1.96%	82.07%
Topic 2	Neg. Rating	49.35%	4.63%	38.06%	12.5%	33.89%	14.72%
	Pos. Rating	4.07%	41.94%	10.28%	39.17%	11.94%	39.44%
Topic 3	Neg. Rating	31.98%	5.31%	25.51%	13.62%	28.86%	6.71%
	Pos. Rating	3.67%	59.03%	7.54%	53.33%	7.87%	56.56%
Topic 4	Neg. Rating	53.01%	2.92%	44.92%	4.8%	46.61%	3.39%
	Pos. Rating	2.17%	41.9%	5.65%	44.63%	4.24%	45.76%
Topic 5	Neg. Rating	40.69%	3.2%	48.2%	10.53%	48.75%	6.65%
	Pos. Rating	2.03%	54.09%	5.54%	35.73%	4.99%	39.61%
Topic 6	Neg. Rating	30.83%	2.5%	28.89%	6.67%	29.17%	6.39%
	Pos. Rating	2.41%	64.26%	5.83%	58.61%	7.5%	56.94%
Topic 7	Neg. Rating	22.95%	4.89%	19.11%	8.6%	16.56%	11.15%
	Pos. Rating	1.59%	70.56%	4.14%	68.15%	3.18%	69.11%
Topic 8	Neg. Rating	29.33%	9.93%	24.14%	14.48%	19.59%	19.59%
	Pos. Rating	2.77%	57.97%	6.9%	54.48%	7.43%	53.38%

*Note.* Neg. Pr. = Negative Prediction; Pos. Pr. = Positive Prediction. Higher values on the diagonal (Neg./Neg. and Pos./Pos.) indicate greater consistency between the scoring of the human raters and the AI models.

**Table 6:** Summary of Classification Performance per Essay Topic. Accuracy (%) and F1 Score Quantify Predictive Performance; Mean Signed Error (MSiE, in Percentage Points) Quantifies Systematic Bias, Where Positive Values Indicate That the AI Model Over-Predicts Positive Ratings Relative to Human Raters.

	Training Data			Validation Data			Test Data		
	Acc.	F1	MSiE	Acc.	F1	MSiE	Acc.	F1	MSiE
Topic 1	96.3	.98	+2.0	90.2	.94	+2.5	90.8	.95	+5.3
Topic 2	91.3	.91	+.6	77.2	.77	+2.2	73.3	.75	+2.8
Topic 3	91.0	.93	+1.6	78.8	.83	+6.1	85.4	.89	−1.2
Topic 4	94.9	.94	+.8	89.6	.90	−.9	92.4	.92	−.9
Topic 5	94.8	.95	+1.2	83.9	.82	+5.0	88.4	.87	+1.7
Topic 6	95.1	.96	+.1	87.5	.90	+.8	86.1	.89	−1.1
Topic 7	93.5	.96	+3.3	87.3	.91	+4.5	85.7	.91	+8.0
Topic 8	87.3	.90	+7.2	78.6	.84	+7.6	73.0	.80	+12.2
Macro *M*	93.0	.94	+2.1	84.1	.86	+3.5	84.4	.87	+3.3
Macro *SD*	3.0	.03	2.3	5.3	.06	2.8	7.4	.07	4.8

*Note.* Acc. = Classification Accuracy (%); F1 = F1 Score; MSiE = Mean Signed Error (FP% - FN%, in percentage points). Positive MSiE indicates AI over-prediction of positive ratings. Macro *M* and *SD* are computed across all eight essay topics

As can be seen, most predictions are close to the human ratings for all essay topics, with accuracy rates (i.e., the rate of correct predictions) usually being above .8 for all essay scores. From this table, we also see that the essay topics differ with regard to their “difficulty,” that is, their probability that a randomly chosen student obtains a positive rating. While positive ratings are more likely for essay topics 1, 3, 6, 7 and 8, we observe more negative ratings for the remaining essay topics. It should be noted that this observation is not interpreted as a violation of fairness. Instead, we interpret it as an indication that the different essay topics are challenging to different degrees.

We are further interested in whether there are groups of essays where the LLM gives systematically higher or lower scores than human raters. In the confusion matrices, such an effect can be detected by investigating the ratio of false positive and false negative predictions, in particular in the validation data set. As we can calculate from Table 5, the ratio of false positive to false negative predictions is between 1.04 and 3.58 for the training data, .85 and 2.1 for the validation data set, and .8 and 3.71 for the test data set. For the validation and test data, positive predictions are more likely than false negative predictions in the essay topics 1, 2, 5, 7 and 8. Three of these topics are among those where positive outcomes are more likely, which might lead to a tendency of the scoring model to give a positive prediction. However, this effect can also be interpreted as a positive bias that affects students working on the mentioned essay topics.

This effect is caused by the general tendency of LLMs to predict classes that occur more frequent in their training data, especially in ambiguous cases where the class membership is unclear. For practical applications, it can still be recommended to use a training set that is mostly representative for the target application of the large language model, if infrequent classes are well enough represented. For an overview on more advanced approaches for addressing imbalanced data, see, for instance, He [72].

Overall, we observe a slight tendency of the AI model to provide more positive scores compared to human raters for some essay topics, which concludes our analysis of fairness.

### Intraclass correlations

For illustrative purposes, we further calculated intraclass correlations ICC(1,1), ICC(2,1) and ICC(3,1) for single and average rating scores, using the notation of Liljequist et al. [33]. These coefficients are estimates of the intraclass correlation derived from three distinct statistical models. ICC(1,1) is based on a one-way random effects model, which assumes the absence of systematic measurement errors (bias). A potential scenario is a reliability study using a single rater, provided there are no procedure-related biases. ICC(2,1) is based on a two-way random effects model. It is used when biases are present and are considered a random effect, such as when the raters are a random sample from a larger population of raters. ICC(3,1) is based on a two-way mixed model. It applies when biases are present but are considered a fixed effect – for instance, when a specific group of raters is selected, and they are the only raters of interest for the study. The values of these intraclass correlations were identical for each topic in the training, validation and test data, but differed between single and average rating scores. Depending on the essay topic, the single rating scores took on values between *ICC* = .47 and *ICC* = .78 for the training data, between *ICC* = .47 and *ICC* = .71 for the validation data, and between *ICC* = .24 and *ICC* = .77 for the test data. For the average rating scores, typically larger values were observed. Depending on the essay topic, the average rating scores took on values between *ICC* = .72 and *ICC* = .91 for the training data, between *ICC* = .74 and *ICC* = .85 for the validation data, and between *ICC* = .48 and *ICC* = .91 for the test data.

Overall, these values indicate a moderate to high interrater reliability on the level of the average rating scores, but poor to moderate interrater reliability on the level of the individual rating scores, following the criteria reported by Koo et al. [34]. However, the analysis of the intraclass correlations implicitly assumes that the same two raters rated all essays for each topic, which seems implausible based on the available description of the data. Given the small number of raters, we did not carry out an analysis of differential rater functioning based on models of IRT.

### Treatment as a regression problem

We now frame the prediction problem as a regression problem, in which we want to predict the specific value between 0 and 1, which is given to each essay, as accurately as possible. Compared to a classification problem, regression models rely on other metrics for assessing their accuracy, such as mean squared errors (MSE), or root of mean squared errors (RMSE). Their estimation is usually also based on different loss functions which consider the changed scale of the predicted variable. In the current implementation, the MSE was used as a loss function for the regression problem. In the following, we demonstrate how switching from a classification to a regression problem affects the evaluation of validity, reliability and fairness.

### Assessing reliability

**Fig 1:**
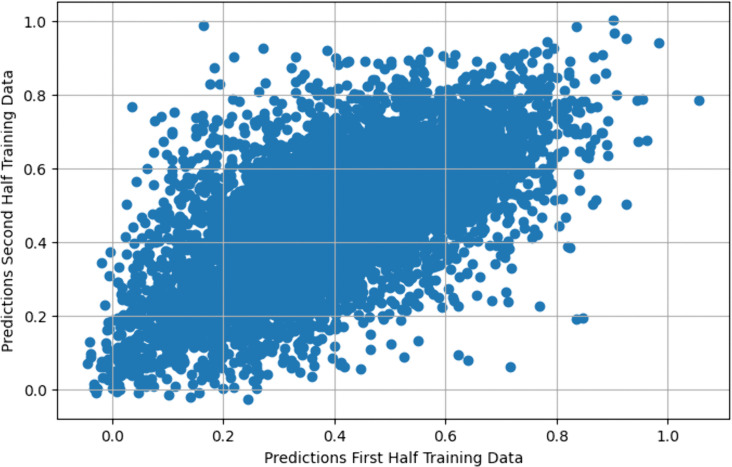
Split-half comparison of essay scores in the training data. Each point compares the predicted score for the first and second half of a text.

**Fig 2:**
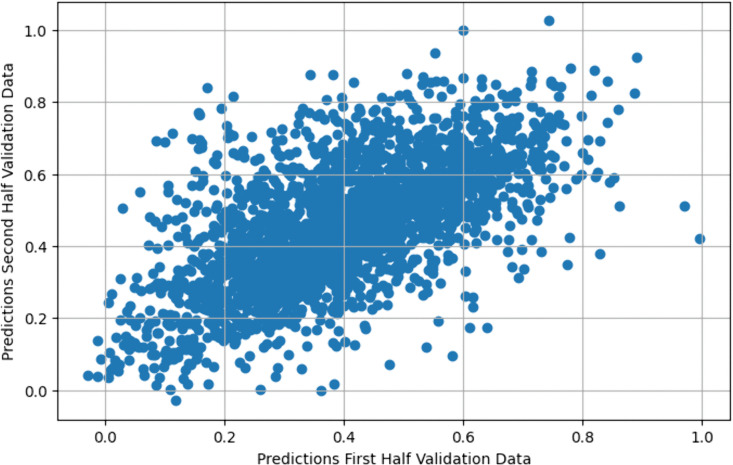
Split-half comparison of essay scores in the validation data. Each point compares the predicted score for the first and second half of a text.

**Fig 3:**
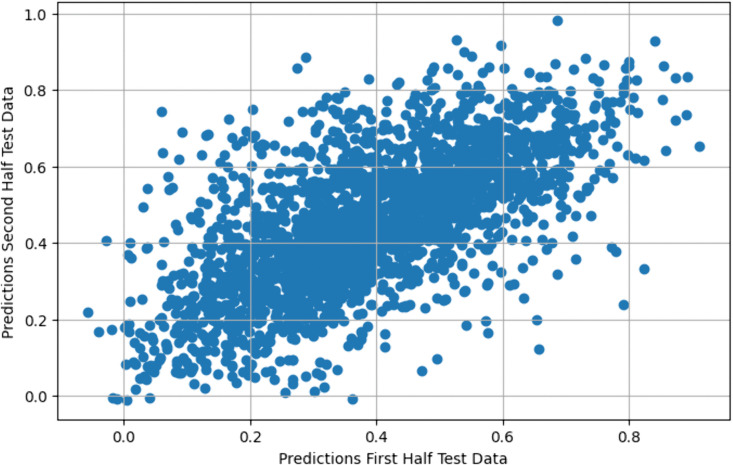
Split-half comparison of essay scores in the test data. Each point compares the predicted score for the first and second half of a text.

As was already outlined, the basic idea of assessing the reliability of AI scores consists in comparing their evaluation and comparison for different parts of the text. In the case of regression problems, these predictions are metric in nature, which leads to the option of calculating a Pearson correlation between these predictions. In our example dataset, we counted the sentences in each text and split them into two halves of similar size, ensuring each half contained only complete sentences. We then calculated a predicted score for each half and measured the correlation between these two scores as an index of reliability. We further calculated confidence intervals (CI) for the correlations using the psych package in R [73]. Short texts consisting of only one sentence were discarded from this analysis. In our example data, this leads to a correlation of *r* = .63 (95% CI: [.61; .65]) in the validation data, *r* = .66 (95% CI: [.65; .67]) in the training data, and *r* = .66 (95% CI: [.64; .68]) in the test data. An application of the Spearman-Brown prophecy formula leads to values of *r* = .77 (95% CI: [.75; .79]) in the validation data, *r* = .80 (95% CI: [.79; .81]) in the training data, and *r* = .80 (95% CI: [.79; .81]) in the test data. Compared to the treatment as a classification model, these numbers are lower, but, depending on the specific application and intended use of the model, might still be satisfactory. These results are further illustrated in the following Figs 1–3.

To improve this measure of reliability, one might further fine-tune the underling model or even change the model. However, given that the quality of the first and second part of an essay might differ significantly, it is plausible that there is an upper bound below 1.0 for the correlation between the predictions for the first and second half.

When applying the regression framework, we did not calculate the intraclass correlation coefficient or apply models based on IRT, since these methods are aimed for the analysis of categorical data.

**Fig 4:**
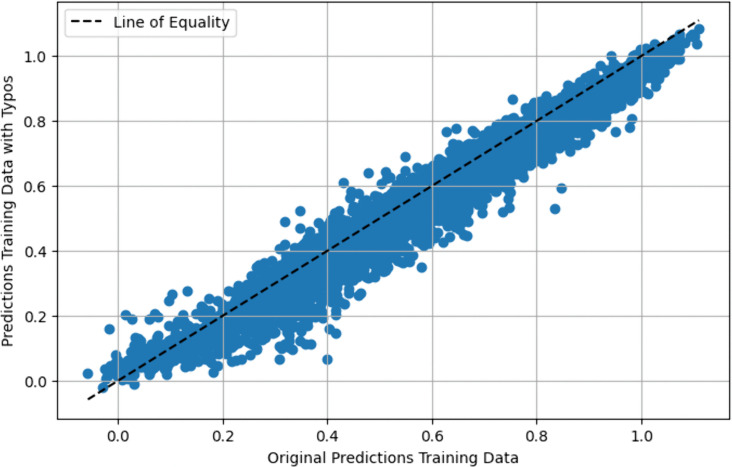
Essay scores before and after inserting 10 random typos, training data. Each text was perturbed by inserting 10 random typos at random positions, and the predictions before and after perturbation are compared.

**Fig 5:**
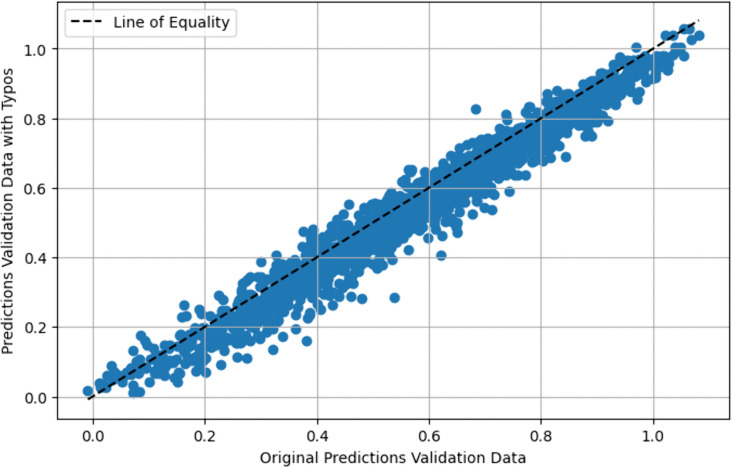
Essay scores before and after inserting 10 random typos, validation data. Each text was perturbed by inserting 10 random typos at random positions, and the predictions before and after perturbation are compared.

**Fig 6:**
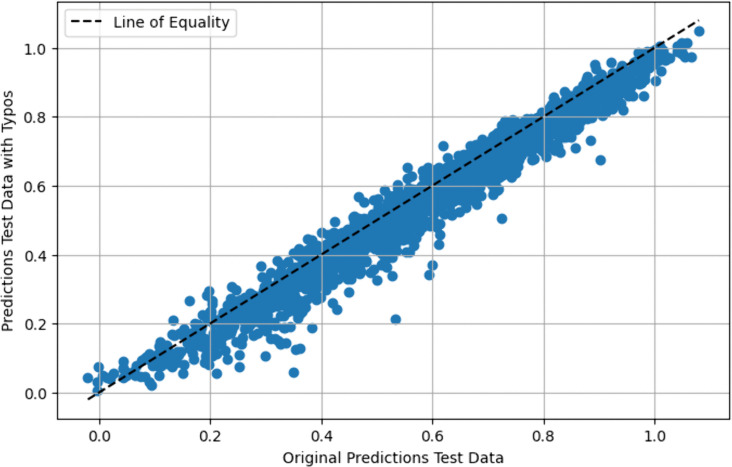
Essay scores before and after inserting 10 random typos, test data. Each text was perturbed by inserting 10 random typos at random positions, and the predictions before and after perturbation are compared.

Again, we can further evaluate the effect of inserting ten typos in the text as an evaluation of its robustness. For the example data set, this evaluation is carried out in the following Figs 4–6 for the training, validation and test data, respectively. In the three data sets, we observe a correlation of about *r* = .98 (95% CI: [.98; .98]) between the predicted scores of texts with and without typos.

As can be seen, the insertion of typos generally reduces the predicted value for the same text, which is in line with theoretical expectations. However, there are a few exceptions, that is, individual texts for which the rating unexpectedly increases. In the Figures above, these texts are visible as dots above the diagonal.

Those unexpected increases, though relatively rare, may be artifacts that stem from the complex, non-linear architecture underlying the neural network. For instance, the insertion of random letters might create a new token (word or word part) that the model associates with higher-scoring texts. This result highlights the nature of the model as a “black box” and also the need of checking the robustness of the model scores.

### Assessing validity

**Fig 7:**
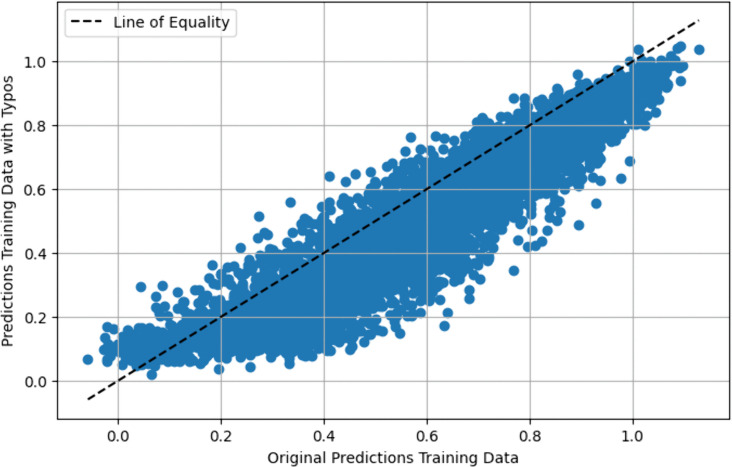
Essay scores before and after inserting 50 random typos, training data. The average score predictions decrease by this change, giving insight into the scoring process.

**Fig 8:**
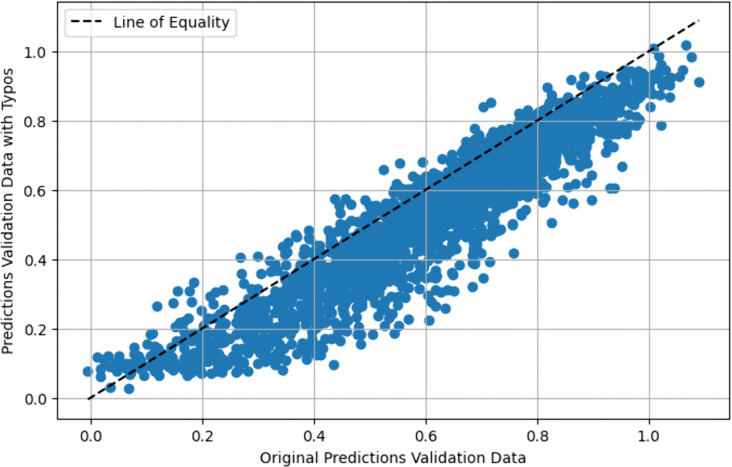
Essay scores before and after inserting 50 random typos, validation data. The average score predictions decrease by this change, giving insight into the scoring process.

**Fig 9:**
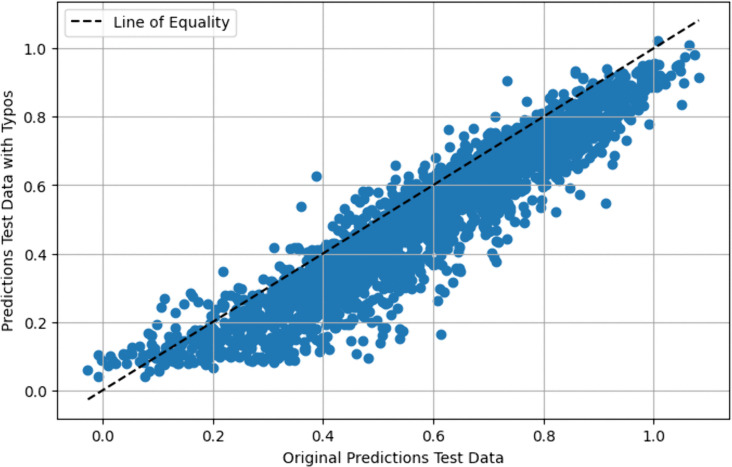
Essay scores before and after inserting 50 random typos, test data. The average score predictions decrease by this change, giving insight into the scoring process.

Based on the results for assessing the robustness of the model scores, we further evaluated a facet of validity by assessing the effect of inserting 50 typos. The results of this evaluation are illustrated in the following Figs 7–9 for the training, validation and test data, respectively.

As can be seen, the insertion of typos generally reduces the predicted value significantly for the same text, which is in line with theoretical expectations. Again, there are individual texts for which the rating increases. In the three data sets, we observe a correlation of about .93 (95% CI in the training data: [.93; .93]; 95% CI in the validation and test data: [.92; .94]) between the predicted scores of texts with and without typos. This correlation is smaller than the analogous correlation observed after the insertion of 10 typos and underlines that the changes to the input texts had a significant effect on the scores.

As with classification problems, we can obtain further evidence for the construct validity of a model’s predicted score by checking its accuracy in the training, validation and test data sets. For the regression model, we obtain a model with an MSE of .01 in the training data and an MSE of .13 in the validation data after training for three epochs.

### Assessing fairness

In the discussion of the evaluation of fairness of AI scores in classification problems we discussed three criteria, which can be directly translated to corresponding criteria for fairness in regression problems: For the first two criteria, we evaluate the metrics of the AI model for different relevant groups of essays, and ensure that the AI scores are a) sufficiently accurate for any group, and b) of comparable accuracy for all groups. As a third criterion, we confirm that c) there are no systematic biases between the predicted and observed scores for any relevant group of essays.

For all eight essay topics, we observed a correlation between the predicted and the observed scores in the range from .83 to .93 in the training data, from .58 to .85 in the validation data and from .65 to .85 in the test data. These numbers are in line with the results of a recent meta-analysis on the agreement of human raters and automated scoring systems [74]. Depending on the intended application of the essay scoring system, they can therefore be interpreted as a satisfactory level of fairness. For biases between the predicted and observed scores, we found mean values between .00 and .03 for all essay topics in the training data, between −.01 and .04 in the validation data, and between −.01 and .04 in the test data. We therefore conclude that there are no systematic biases in the predictions.

## Discussion

**Fig 10:**
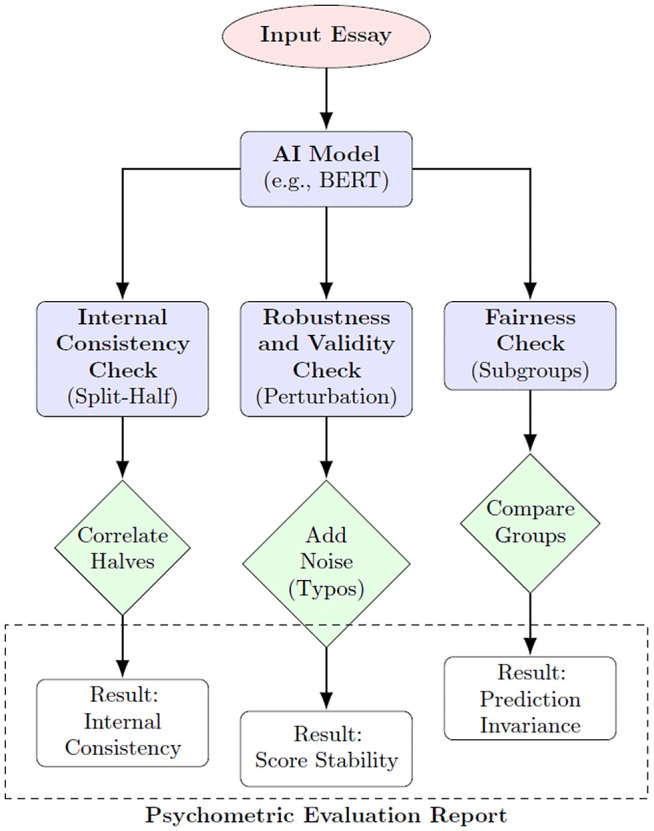
Schematic Overview of the Proposed Evaluation Framework. The diagram illustrates how a single essay input is processed through three parallel evaluation pipelines to evaluate Internal Consistency, Robustness/Validity, and Fairness.

We have discussed three central standards for psychological and educational assessments, and proposed specific methods for how to evaluate them for numerical scores that were obtained using models of artificial intelligence. This led to an evaluation protocol that is evaluated in Table 1. This protocol was illustrated via an empirical application. In this application, we used an LLM to predict human ratings. This problem was framed as a classification or regression problem, which led to slight differences in the proposed evaluation methods. Compared to existing testing frameworks [29,36,75], we focused on providing specific statistical evaluations that are aligned with psychometric standards. The workflow is illustrated in Fig 10.

In our example, the numerical scores of all essays were obtained using a specific model architecture, namely the encoder-based transformer model DistilBERT. In principle, such scores can be obtained by multiple methods from the field of natural language processing (NLP), such as bag-of-words models, recurrent neural networks or even generative transformer models such as GPT-4o or Gemini, which are based on the evaluation of textual prompts. For the proposed methods, the specific nature of the underlying NLP model only plays a role in that it affects how the proposed methods can be technically implemented. On a conceptual level, the prediction model is treated as a “black box,” and the proposed methods focus on their predictions. This characteristic also means that we cannot check all aspects of fairness, reliability and validity that are generally evaluated in, for instance, an IRT framework. For instance, we generally cannot check our black box models for structural invariance, which is related to differential item functioning and measurement invariance in a FA and IRT framework. Furthermore, our empirical results are specific to DistilBERT and the Hewlett Foundation dataset, and may not generalize to other models, domains, or corpora. This slight conceptual weakness is compensated by a strong independence of the discussed methods from the underlying measurement framework.

If significant violations of reliability, validity or fairness are detected in an essay scoring model, this usually indicates that a modification of the model is required. As a first step, longer training or the fine-tuning with additional data might lead to a sufficient improvement of the model. However, in case that additional training or fine-tuning does not resolve these problems, it might be necessary to switch to a different model instead.

For the evaluation of automated essay scoring systems, such as those considered in this text, we can make recommendations how the presented methods should be included in existing evaluation pipelines. First, we recommend that evaluations of such systems report at least one measure of internal consistency (such as a split-half analysis) and a measure of robustness to minor changes. Validity arguments for such systems should at least be supported by empirical evidence of strong predictive accuracy against human scores. These arguments can be further supported by additional methods, such as methods of interpretable machine learning, when this is technical feasible and provides meaningful evidence. For instance, when the essay scoring systems aims at checking the spelling of a text, methods of interpretable machine learning should be able to detect mis-spelled words. Fairness arguments should, at a minimum, compare predictive accuracy and mean score differences across relevant, protected demographic subgroups, while clearly acknowledging any dataset limitations.

### Interpretation of the empirical findings

In our empirical example, we found evidence for high internal consistency (Spearman-Brown correlations > .7) and robustness and several important aspects of validity. The reported confidence intervals demonstrate the stability of these results. In the data set used for the demonstration, the automated essay scoring data set of the Hewlett Foundation, we used essay topics as a proxy variable for the evaluation of fairness. The presented framework provides tools for evaluating fairness aspects with regard to gender, education and similar covariates if those are available.

### Ethical Implications and Transparency

It is important to note that passing the checks outlined in this framework does not equate to the ethical safety of a system for generating scoring or other feedback, which is particularly relevant in high-stakes educational applications. The “black box” nature of deep learning models poses significant challenges for accountability. This is particularly relevant for the evaluation of fairness, where procedural fairness needs to be covered outside of the statistical evaluation provided here.

In the holistic data example used in this illustration, values of explainable AI such as SHAP values can be noisy, since it is impossible to relate the overall outcome to individual tokens. Instead, systematic experiments based on varying model input can allow an evaluation of the model’s behavior.

## Outlook and limitations

The evaluation framework presented in this work has some limitations, but also points to possible future extensions and areas for future research. A limitation of the current empirical illustration is that methods of explainable AI were not applied, as the holistic scoring criterion did not lend itself to token-level interpretation. Future work with more specific scoring rubrics could demonstrate these methods empirically.

Topically, we focused on the specific application of automated essay scoring, and while the presented methods can help to evaluate specific applications of essay scoring models, they do not allow a relative comparison of various essay scoring systems that is independent of their application. For instance, we are not able to conclude based on this techniques which model among a set of given LLMs, such as GPT-4o or Gemini, is best suited for scoring human essays. An interesting field of future research is the construction of a benchmark set of essays which could be used to compare essay scoring systems against each other. Such a set of essays could contain typical essays from various settings (such as schools and universities) and, possibly, various languages, but also essays that contain typical flaws and errors (in various aspects such as spelling, argumentation, text consistency, or grammar) that should be detected by essays scoring systems.

A second extension concerns the development of similar tools and guidelines for models that provide non-numerical output. For instance, generative AI models such as GPT-4o can be instructed to provide verbal feedback on automated essays. Compared to BERT and similar models, evaluating the robustness of the output of these model could include an evaluation of its robustness to changes in the prompt. In contrast to the methods outlined above, such an extension would require methods that are more qualitative in nature while evaluating aspects of reliability, validity and fairness. A general approach for handling verbal output could consist of using numerical linguistic features to quantify specific characteristics of the texts, such as readability, which can be quantified by formulas based on the average word and sentence lengths (e.g., the Flesch–Kincaid readability tests). A related idea is using LLMs to get a rating of the emotional tone of a text or any feature that is of interest. Evidence for the validity of these quantifications can be provided by human inspection.

A third direction for possible extensions could develop similar methods for the evaluation of AI models that evaluate non-verbal responses. A possible use case could be AI models that evaluate drawn figures (e.g., as part of educational or neuropsychological assessments) or videos.

## Conclusion

If AI models are used as part of psychological and educational assessments, they must be held to rigorous professional standards. In this work, we moved beyond conceptual discussion and suggested an evaluation framework and protocol for validating AI-based scores.

Our methodological contributions are threefold. First, we combined methods based on split-half analysis with input perturbation to assess aspects of reliability, namely internal consistency and robustness. Second, input perturbation can also serve as a critical stress test for construct validity. For instance, a model that delivers similar outputs despite massive text changes fails to measure writing quality properly. Third, we outlined a fairness check that compares the prediction accuracy across subgroups and which was applied to essay topics as a proxy variable.

Our empirical example provided evidence for high internal consistency (Spearman-Brown correlations > .7), robustness and several important aspects of validity. We also illustrated the evaluation of aspects of fairness by comparing the accuracy of the machine learning prediction across several relevant subgroups.

Under this perspective, we recommend that future deployments of automated essay scoring systems adopt the checklist provided in Table 1 or a similar evaluation system. While AI models can be proficient in score prediction, fairness and validity are not inherent properties of the architecture and must be verified through rigorous, standardized psychometric evaluations. In this paper, we provide some suggestions for such evaluations, but feel that the development of further methods is necessary. Future work should develop standardized benchmark corpora for psychometric applications and extend these methods to non-numerical AI output, as discussed above.

## Supporting information

S1 Appendix List of essay prompts. Descriptions of the eight essay prompts of the Hewlett Foundation automated essay scoring data set used in the empirical example.
(PDF)
