# Supplementary material for: Testing standards for AI-based scores in automated essay scoring
Source: PLoS One. 2026 Jul 31;21(7):e0354680. doi: 10.1371/journal.pone.0354680 (PMC13426959; doi:10.1371/journal.pone.0354680)
Supplement: S1 Appendix — Descriptions of the eight essay prompts of the Hewlett Foundation automated essay scoring data set used in the empirical example. (PDF) [file pone.0354680.s001.pdf]

S1 Appendix: List of Essay Prompts

Here is a list of all prompts used in the empirical example:

- In Essay Group 1 (1783 essays, about 350 words on average), students were asked to write a persuasive letter to a local newspaper in which they state their opinion on the effects computers have on people. The aim was to persuade the readers to agree with the letter authors.
- In Essay Group 2 (1800 essays, about 350 words on average), students were asked to write a persuasive letter to a newspaper that reflects their views on censorship in libraries. The presented position should be supported with convincing arguments from their own experience, observations, and/or readings.
- In Essay Group 3 (1726 essays, about 150 words on average), students were first presented with a source essay titled "Rough Road Ahead: Do not exceed posted speed limit" by Joe Kurmaskie, which narrates a story about a cyclist. They were then asked to write a response that explains how the features of the setting affect the cyclist, and which includes examples from the essay that support the conclusion.
- In Essay Group 4 (1770 essays, about 150 words on average), students were first presented with a source essay titled "Winter Hibiscus" by Minfong Ho. They were asked to write a response that explains why the author concludes her story with the given last paragraph, including details and examples from the story to support these ideas.
- In Essay Group 5 (1805 essays, about 150 words on average), students were first presented with a source essay titled "Narciso Rodriguez" by Narciso Rodriguez. They were then tasked to describe the mood created by the author in the memoir, and to support their answer with relevant and specific information from the text.
- In Essay Group 6 (1800 essays, about 150 words on average), students were first presented with a source essay titled "The Mooring Mast" by Marcia Amidon Lüsted, which is a text on the construction of the Empire State Building. They were asked to describe the obstacles that the builders of the Empire State Building faced in their attempts to allow dirigibles to dock there, and they were requested to support their answers with relevant and specific information from the text.
- In Essay Group 7 (1569 essays, about 250 words on average), students were tasked to write a story about the topic of patience, i.e., a story when they themselves or someone they knew were patient, or a story in which they write about patience in their own way.
- In Essay Group 8 (723 essays, about 650 words on average), students were tasked to write a true story in which laughter was one element or part.
